# Successful Aging: A Psychosocial Resources Model for Very Old Adults

**DOI:** 10.1155/2012/934649

**Published:** 2012-07-26

**Authors:** G. Kevin Randall, Peter Martin, Mary Ann Johnson, Leonard W. Poon

**Affiliations:** ^1^CC Wheeler Institute, Bradley University, 05 Bradley Hall, 1501 W. Bradley Avenue, Peoria, IL 61625, USA; ^2^Gerontology Program, Iowa State University, 1096 LeBaron Hall, Ames, IA 50011-1120, USA; ^3^Department of Foods & Nutrition, The University of Georgia, 143 Barrow Hall, 115 DW Brooks Dr, Athens, GA 30602, USA; ^4^Institute of Gerontology, College of Public Health, The University of Georgia, 255 E. Hancock Avenue, Athens, GA 30602-5775, USA

## Abstract

*Objectives*. Using data from the first two phases of the Georgia Centenarian Study, we proposed a latent factor structure for the Duke OARS domains: Economic Resources, Mental Health, Activities of Daily Living, Physical Health, and Social Resources. *Methods*. Exploratory and confirmatory factor analyses were conducted on two waves of the Georgia Centenarian Study to test a latent variable measurement model of the five resources; nested model testing was employed to assess the final measurement model for equivalency of factor structure over time. *Results*. The specified measurement model fit the data well at Time 1. However, at Time 2, Social Resources only had one indicator load significantly and substantively. Supplemental analyses demonstrated that a model without Social Resources adequately fit the data. Factorial invariance over time was confirmed for the remaining four latent variables. *Discussion*. This study's findings allow researchers and clinicians to reduce the number of OARS questions asked of participants. This has practical implications because increased difficulties with hearing, vision, and fatigue in older adults may require extended time or multiple interviewer sessions to complete the battery of OARS questions.

## 1. Introduction

Aging is often conceptualized as a developmental challenge to maintain balance between the gains and losses of resources necessary for adaptation to age-related change, with losses increasing over the lifespan [1, 2]. Yet, Von Faber and colleagues [3] reminded us that, “Successful aging as an optimal state implicates more than physical well-being and fits the World Health Organization's definition of health as a state of complete physical, mental, and social well-being and not merely the absence of disease or infirmity.” These resources-material, social, or personal characteristics essential for successful aging-hold a prominent position in studies of older adults [4–6]. Consequently, the need for a valid, reliable, and efficient resource measure, designed for use in clinical and community settings with older adults, arose. In response, Fillenbaum [7] and associates developed the *Multidimensional Functional Assessment of Older Adults: The Duke Older Americans Resources and Services Procedures* (OARS hereafter). Since development of the OARS, advances in statistical methodology, computer technology, and software programs have made factor analytic procedures commonplace, enabling researchers to suggest less complex and shorter versions of measurement scales and to model measurement error in empirical studies. To date, we know of no studies that investigated the underlying, latent factor structure of the five OARS resources with data from older adults—the purpose of the present study.

Studies on multiple resources and successful aging among older adults have grown tremendously [8–11], spawning psychometric concerns regarding how and what to assess [12]. Researchers have demonstrated the need to expand investigations of multidimensional resources in older populations [13, 14]. This body of work was motivated not only by the increased prevalence of older adults but also by a growing concern among those assessing and caring for frail older adults. The OARS was developed by a multidisciplinary team who recognized that older adults' personal well-being encompasses many aspects or multiple functions [7]. Because older adults often present with chronic disabilities or ailments [15, 16], the developers of the OARS designed an assessment tool that focused primarily on adaptation and the maintenance of personal well-being in five resources: Social Resources, Economic Resources, Mental Health, Physical Health, and self-care capacity or functional health (including both instrumental activities of daily living (IADL) and activities of daily living (ADLs)). This instrument has received widespread use by a diverse group of geriatric practitioners, researchers, and service group providers such as epidemiologists characterizing particular populations, clinicians assessing patient status, resource allocators providing services, and program evaluators investigating the impacts of interventions [12, 17–19].

In addition, because clinical work and empirical research may be tiring and confusing for older participants, a reduced version of the five OARS resources as modeled by five latent variables would prove helpful in reducing the time required to assess older adults' resources. However, few studies have specified the OARS resources as latent variables [20–22]. Further, to date, no study was found that developed a measurement model for all five OARS resources.

The purpose of this study was to specify and test the latent factor structure of the five OARS resources administered to participants in their 60s, 80s, and 100s. We hypothesized that (a) the resource model proposed by Fillenbaum [7] can be obtained using data from old and very old individuals and (b) a reduced short version of the OARS will yield a satisfactory latent variable solution.

## 2. Methods

### 2.1. Procedure and Participants

 Participants were selected through the assistance of the University of Georgia Survey Research Center, the Office of the Governor of Georgia, the media, and local older adult service organizations [23–25]. Selection criteria for the final sample of community-dwelling individuals included a score of 23 or higher on the Mini-Mental Status Examination (MMSE; [26]) or a score of 2 or lower on the Global Deterioration Scale [27].

The data collection at phase one included 321 older adults (217 women, 104 men), classified as sexagenarians (*n* = 91), octogenarians (*n* = 93), and centenarians (*n* = 137). At time two, 201 participants provided data for this longitudinal study: 70 sexagenarians, 63 octogenarians, and 68 centenarians. Those in their 60s and 80s were followed up within 60 months; due to mortality attrition, centenarians were followed up within 20 months. Almost one-third of the sample was African American (27.7% and 30.8% at Time 1 and Time 2, resp.). The majority of the sample was female (67.6%), well-educated (at least graduated from high school), and rated their health as excellent or good (Table 1). As noted earlier, in this sample of old and very old adults, mortality attrition resulted in a reduction of participants, particularly among the centenarians, from 321 at time 1 to 201 at time 2.  Table 2 examines the differences for the OARS manifest variables and two measures of cognitive status. Overall, the sample at time 2 showed significantly higher scores relative to time 1.

### 2.2. Measures

Because the purpose of this study was to develop a latent variable model for the five OARS resources, in this section we provide a brief overview of the measures based upon Fillenbaum's [7] work. Details of our final latent variables and corresponding indicators are presented in Section 3. In addition, the appendix lists each resource and its indicators and associated questions, based on our final results.

#### 2.2.1. Economic Resources

Six questions were asked; examples included “How well does the amount of money you have take care of your needs—very well, fairly well, or poorly?” and “Please tell me how well you think you are now doing financially as compared to other people your age—better, about the same, or worse?” These items were scaled from 0: poorly or worse to 2: very well or better.

#### 2.2.2. Mental Health

Satisfaction (six items), sleep disturbance (two items), lethargy (6 items), and paranoid (three items) comprised the four dimensions of Mental Health [7]. Examples of questions included (a) satisfaction: “In general, do you find life exciting, pretty routine, or dull?” scaled so that 0: dull, 1: pretty routine, and 2: exciting; (b) sleep disturbance: “Do you wake up fresh and rested most mornings?” scaled so that 0: no, 1: yes; (c) lethargy: “Have you had periods of days, weeks, or months when you couldn't take care of things because you couldn't “get going?” scaled so that 0: no, 1: yes; (d) paranoid: “Does it seem that no one understands you?” scaled so that 0: no, 1: yes.

#### 2.2.3. ADLs

Two commonly used dimensions, instrumental activities of daily living (IADL; seven items) and physical activities of daily living (PADLs; six items), comprised the self-care capacity assessment. An example of an IADL question included “Can you do your housework?” scaled so that 2: without help (can clean floors, etc.); 1: with some help (can prepare some things but unable to cook full meals yourself) or 0: are you completely unable to prepare any meals? A PADL question was “Can you dress and undress yourself” scaled so that 2: without help (able to pick out clothes, dress and undress yourself) 1: with some help, or 0: are you completely unable to dress and undress yourself?

#### 2.2.4. Physical Health

Three questions assessing subjective self-rated health were included. For example, participants responded to “How would you rate your overall health at the present time—excellent, good, fair, or poor?” scaled so that 0: poor and 3: excellent.

#### 2.2.5. Social Resources

Social Resources were measured using seven questions. An example of a question focusing on the interaction aspect of social support was “How many people do you know well enough to visit with in their homes?” Participants chose from a scale of 0: none to 3: five or more. An assessment of dependability of social support included two questions. These questions were answered 1: yes and 0: no. For example, one question was “Do you have someone you can trust and confide in?” A third assessment of the affective domain of social support also included two questions, scaled 1: yes and 0: no. For example, one question was “Do you see your relatives and friends as often as you want to or not?”

### 2.3. Data Analysis

The analyses conducted to confirm a measurement model included the following steps for each resource: (a) specifying and testing Fillenbaum's subscales; (b) adapting Fillenbaum's recommendations when modeling difficulties were encountered; (c) employing exploratory factor analyses (i.e., principal axis factoring retaining three factors with oblique rotation) to assess relationships within the data and to posit possible indicators for latent constructs; (d) testing the measurement model by confirmatory factor analyses. We developed at least three indicators for each resource and then tested the latent variable measurement model via confirmatory factor analysis.  Table 3 provides assessment of measurement model fit, standardized loadings, and the uniqueness or *R* ^2^ for each indicator. All variables were scaled so that higher scores indicated higher levels of the resource. In the appendix, we provide a nontechnical summary of our results, listing the recommended questions for each indicator of the five OARS resources.

We used full-information maximum likelihood (FIML) to estimate our models [28, 29]. For our latent variable analyses we used *M*plus 6.11 [30] and employed FIML with the estimator MLR (maximum likelihood parameter estimates with standard errors and a mean-adjusted chi-square test statistic that are robust to nonnormality).

Exploratory factor analyses were conducted using SPSS 18.0, whereas confirmatory factor analyses and structural equation modeling were conducted with *M*plus [30]. Overall model fit was assessed by employing the Satorra-Bentler chi-square test statistic. This type of chi-square test statistic provided maximum likelihood parameter estimates with standard errors and a mean-adjusted chisquare test statistic that is robust to nonnormality of measures. Because the chisquare goodness of fit test and its corresponding probability value are sensitive to sample size, often making it difficult to accurately assess model fit when limited to this single statistic [31, 32], other measures of model fit were reported including the Comparative Fit Index (CFI) [33], the Tucker-Lewis coefficient (also called the NNFI) (TLI) [34], Browne and Cudeck's [35] root mean squared error of approximation (RMSEA), and the standardized root mean squared residual (SRMR). It has been suggested that values close to .95 for TLI and CFI, .08 for SRMR, and .06 for RMSEA are necessary before concluding that a relatively good fit between the observed data and the hypothesized model exists [36, 37].

## 3. Results

*Economic Resources* included six items. We conducted an exploratory factor analysis (principal axis factoring) with an oblique rotation and extracted three factors, accounting for 76 percent of the variance. Based upon these results, we constructed three indicators. First, we summed the three dichotomous items tapping the sufficiency of the respondent's economic resources to meet emergencies and provide extras currently and in the future (Cronbach's alpha = .81). This indicator, *Sufficient Income*, was recorded so that the scores ranged from 0 to 2. (Because we summed these indicators to create a manifest variable in other analyses, we recorded the indicators for equal weighting.) Second, two items loaded on a second factor, both asking the respondents how well they were doing financially. These two items were then averaged to create a second indicator, *Overall Income*, and assessed how well the respondents felt they were doing relative to their overall financial well-being (Cronbach's alpha = .59). Finally, the last item, *Meet Payments*, assessed the participant's expenses, was recorded to 0–2, and used as a third indicator. This item tapped the respondents, ability to meet payments. The three indicators loaded significantly and substantively on the latent variable, Economic Resources, at Time 1 and Time 2 (Table 3).

Inspection of the *Mental Health* assessment revealed that five items had 90% or more respondents scoring alike, five had 80% or more of respondents scoring alike, and three had 70% or more of respondents scoring alike. Thus, we did not use these items and specified a latent variable with three single-item indicators: (a) “In general, do you find life exciting, pretty routine, or dull?” (b) “How would you rate your mental or emotional health at the present time?” and (c) “Taking everything into consideration how would you describe your satisfaction with life in general at the present time?” These three indicators, *Exciting*, *Overall Mental Health*, and *Life Satisfaction*, loaded significantly and substantively on the latent variable Mental Health at Time 1 and Time 2 (Table 3).

*Activities of Daily Living* (IADL) consisted of instrumental activities of daily living (seven items) and physical activities of daily living (six items). Because descriptive statistics demonstrated that for those in their 60s and 80s few difficulties with physical activities of daily living were encountered, we did not include this subscale but created three indicators for IADL based upon an exploratory factor analysis of the seven items of IADL. The first indicator, labeled *Getting Out*, included items assessing (a) “Can you use the phone?” (b) “Can you get to places out of walking distance?” and (c) “Can you go shopping for groceries or clothes?” Cronbach's alpha for these three items was .76. The second indicator, *Housework*, was comprised of three items (Cronbach's alpha = .88) assessing: (a) “Can you prepare your own meals?” (b) “Can you do your own housework?” and (c) “Can you handle your own money?” The third indicator, *Medicine*, was a single item, “Can you take your own medicine?” The three indicators loaded significantly and substantively on the latent variable IADL at Time 1 and Time 2 (Table 3).

*Physical Health* consisted of three single-item indicators assessing subjective health perceptions. First, for the indicator *Low Troubles*, respondents were asked, “How much do your health troubles stand in the way of your doing the things you want to do?” Second, the question for the indicator *Overall Health* asked “How would you rate your overall health at the present time?” A third question for the indicator *Comparative Health* asked, “Is your health now better, about the same, or worse than it was five years ago?” These three indicators loaded significantly and substantively on the latent variable Physical Health at Time 1 and Time 2 (Table 3).

*Social Resources* included seven items. Because three dichotomous items did not provide much variance (over 90% of respondents scored “yes”), we did not use them. We then conducted an exploratory factor analysis on the remaining questions. Four items were used in our initial latent variable: (a) “How many times did you talk to friends, relatives, or others on the phone in the past week?” (b) “How many people do you know well enough to visit in their homes?” (c) “How many times in the past week did you spend some time with someone who does not live with you?” and (d) “Do you find yourself feeling lonely?” (recorded so that high scores reflected low loneliness). Thus, a latent variable was specified with *Phone Talk* as the first indicator, with *Visit Network Number *as a second indicator, *Visits With Others* as a third indicator, and *Loneliness* as a fourth.

Based on the previous exploratory factor analyses, we specified and tested a measurement model using confirmatory factor analysis and comprised of the five factors and their respective indicators as previously discussed. However, in the first test of the measurement model, loneliness did not significantly load on the latent variable for Social Resources at Time 1 (*t* = 1.62); this item was dropped and not used as an indicator in further analyses. Next, we conducted a similar analysis and these indicators loaded on the latent factor, Social Resources, significantly and substantively at Time 1 (see Table 3 for the results). However, at Time 2 the second and third indicators did not load significantly or substantively, although the overall measurement model fit to the data was adequate. This indicates that the construct might have changed over time, and the results for Time 2 Social Resources need to be stated with caution (Table 3).

The five latent variables were significantly correlated with one another at each measurement occasion with a few exceptions at Time 2 (Table 4). For example, at Time 2 Social Resources was only significantly associated with IADL; also, Physical Health was not significantly associated with either Mental Health or Social Resources at Time 2. Also, power issues may have influenced the results as the sample size was *N* = 321 at time 1 and *N* = 201 at Time 2, resulting in a lack of findings that may have existed. For example, consider the correlation between Social Resources and Mental Health at Time 2: *r* = .25, *P* > .05. However, at Time 1, with the larger sample size, two correlations close to the same magnitude as that between Social Resources and Mental Health at Time 2—the correlation between Economic Resources and IADL (*r* = .25, *P* ≤ .01) and the correlations between Economic Resources and Social Resources (*r* = .27, *P* ≤ .01)—are significant, indicating a likely reduction in power at Time 2.  Table 5 presents the final correlations and descriptive statistics for all indicators of the OARS measurement model.

Finally, we attempted a nested model test for factorial invariance over time by constraining the loadings of each indicator at Time 1 equal to the same indicator at Time 2. Also, the residual for each indicator at Time 1 was correlated with its counterpart at Time 2. This constrained or nested model did not fit the data well: MLR *χ* ^2^(354, *N* = 201) = 527.03, *P* = .001, CFI = .91, TLI = .89, RMSEA = .05, and SRMR = .07. However, despite increasing the number of iterations and specifying starting values, we were not able to get the unconstrained or base model to converge. This measurement model included the Social Resources latent construct at Time 2 that did not have significant loadings for its estimated indicators. Thus, the overall poor model fit may be due to a poor measurement model for Social Resources.

As a follow-up, we decided to fit a measurement model without the Social Resources latent variable. The unconstrained or base model, including the four latent variable resource constructs (without Social Resources at Time 1 and Time 2), specified with correlated residuals across time, fit the data well: MLR *χ* ^2^(211, *N* = 201) = 289.09, *P* = .001, CFI = .95, TLI = .94, RMSEA = .04, and SRMR = .06. Next, we added across time constraints to the factor loadings for each corresponding indicator and ran the model. This constrained or nested model with eight more degrees of freedom fit the data well also: MLR *χ* ^2^(219, *N* = 201) = 300.18, *P* = .001, CFI = .95, TLI = .94, RMSEA = .04, and SRMR = .07. Finally, we performed a nested model chi-square difference test following the specifications provided by B. Muthén and L. Muthén [30] for the MLR chi-square. In this case, the chi-square difference was 11.06 with eight degrees of freedom, *P* = .20. Thus, no significant difference was found between these two models and it is reasonable to assume factorial invariance over time.

## 4. Discussion

The focus of this study was a widely used integral part of the *Multidimensional Functional Assessment of Older Adults: The Duke Older Americans Resources and Services Procedures* [7]. To date, few studies have specified one or more of the five OARS resources as latent variables [20–22] and we found no studies with older adults, especially centenarians, specifying all five OARS resources as latent variables. Thus, empirical and clinical attention to multidimensional assessments of older adults and their successful aging, especially those in their 80s and 100s, and the continued popularity of the OARS instrument as a standardized scale motivated this study development of a measurement model of psychosocial resources essential to successful aging for very old adults.

Five latent variables with three indicators each, corresponding to the five OARS resources, were specified; the combined measurement model fit the data adequately using a sample of older adults in their 60s, 80s, and 100s. Three results from the measurement analyses are noteworthy. First, because of advances in SEM programs and techniques allowing specification of measurement error and latent factor modeling, a comprehensive measurement model of the five OARS resources would prove useful to researchers and those assessing and caring for older adults. With the exception of Social Resources at Time 2, researchers employing the OARS may confidently specify the measurement model tested in this study. The measurement model tested in this study fit the data well at Time 1 and Time 2. Factor loadings (except those of Social Resources at Time 2) were significant and substantive, providing adequate evidence of an acceptable latent variable measurement model for the five OARS resources.

Second, supplementary analyses of factorial invariance over time, conducted without Social Resources at Time 2, revealed that in this sample, the four latent variables (i.e., Economic Resources, IADL, Physical Health, and Mental Health) fit the data well and did not change significantly over time. It is noted that for the younger participants (i.e., those in their 60s and 80s), five years elapsed between measurement occasions, whereas, for the centenarians, 20 months. Researchers interested in developmental questions of change over time are encouraged to employ the measurement model verified by this study and to extend this work by carefully considering the time intervals necessary for proximal and distal influences to unfold among older adults.

Third, Social Resources tended to have the lowest loadings per indicator. In this sample, relative to the other indicators, talking on the phone is the main indicator tapping the respondent's Social Resources. Burholt and colleagues [38] investigated OARS Social Resources in a population-based study of older adults living independently in six Western European countries and argued that the items demonstrated a breadth of conceptual assessment. It is our contention that such is the case with the three single-item indicators for the latent variable Social Resources. These indicators may assess a breadth of structure and at times the items may not be related. These three items may better serve as a checklist of social resources structure. Expecting relatively high factor loadings for such a construct may be unfounded (see [39], for a discussion regarding why checklist assessments often exhibit low internal consistency). Thus, other valid and reliable assessments of social support might be examined to augment or supplant the items included in the OARS assessment. Finally, empirical work investigating the relationship between measures of social support and loneliness has demonstrated discriminant validity; despite the strong association these measures tap different constructs [40, 41]. This is consistent with the finding of this study; the loneliness item did not load significantly on the Social Resources latent variable and was not included in the final measurement model.

### 4.1. Limitations and Direction for Future Research

Several limitations, however, exist that affect the generalization of this study's results. First, the participants were Southeastern older adults in reasonably good health, mentally competent, and community-dwelling. Second, the younger age groups (those in their 60s and those in their 80s) were randomly selected by race and gender to approximate older adults in Georgia. However, in contrast, centenarians were selected using convenience sampling through state and local agencies. Also, the sexagenarians and octogenarians were assessed in testing locations; centenarians completed their assessments at home. In addition, for the two younger age groups, measurement occasions were five years apart, but for the centenarians the measurement occasions were approximately 20 months apart. With only two waves of data, longitudinal results are to be interpreted with caution. Future research will want to employ other valid assessments of similar assessments of the five resources for comparison, particularly with larger, more homogeneous samples. This would provide opportunity to compare the relationships between the revised OARS latent resources based on our results and other known measures. Also, using a more homogenous sample of older adults might mitigate some of the methodological difficulties inherent in our heterogeneous sample that includes three age groups of old (60s and 80s) and very old adults (100s).

Finally, the items used for Social Resources in this study could be improved in future research. All the latent variables except Social Resources were fairly consistent across measurement occasions. In fact, for the models tested without the Social Resources latent variable, the overall measurement model fit the data well and factorial invariance of the latent variables over time was substantiated. Future research is encouraged to consider other measures of Social Resources (see [42], for 12 different measures assessing social support).

This study employed data from the Georgia Centenarian Study and used the popular Duke OARS [7] to develop a measurement model consisting of latent variables for Economic Resources, Instrumental Activities of Daily Living, Physical Health, Mental Health, and Social Resources. The model was specified and affirmed using longitudinal (two waves) data from a sample of sexagenarians, octogenarians, and centenarians, further substantiating the robustness of these latent variables in research with older adults. The results of this study allow reduction of the numerous items used in assessing the five OARS resources. This has valuable and practical implications because increased difficulties with hearing, vision, and fatigue in older adults may require extended time or multiple interviewer sessions to complete the extensive battery of questions in the OARS. Thus, researchers conducting etiological investigations, health professionals conducting intake and out-patient assessments, and other practitioners wishing to employ the OARS with older populations and the resources essential to successful aging will benefit from using this reduced version.

## Figures and Tables

**Table 1:** Sample demographic characteristics.

Variables	Time 1		Time 2		*χ* ^2^
	*n*	%	*n*	%
Sex					1.59
Male	104	32.4	60	29.9	
Female	217	67.6	141	70.1	

Race					2.61
Black	89	27.7	62	30.8	
White	232	72.3	139	69.2	

Age group					18.86^∗∗∗^
60s	91	28.3	70	34.8	
80s	93	29.0	63	31.3	
100s	137	42.7	68	33.8	

Education					6.19
0-8 years	90	28.2	55	28.8	
High school	84	26.3	42	22.0	
Business/trade school	23	7.2	14	7.3	
College	75	23.6	44	23.0	
Graduate school	47	14.7	36	18.8	

Self-rated health					4.7
Excellent	67	21.1	39	20.2	
Good	159	50.2	93	48.2	
Fair	79	24.9	52	26.9	
Poor	12	3.8	9	4.7

Because of rounding, percentages may not add to 100.

^∗∗∗^ *P* < .001.

**Table 2:** Differences between participants at Time 1 and participants at Time 1 and Time 2.

Variables	Time 1 Only		Time 1 and Time 2		*t*
	*M*	SD	*M*	SD
Mini-Mental (MMSE)	25.30	2.72	26.31	3.01	−3.03^∗∗^
Short Portable (SPMSQ)	8.63	1.58	9.04	1.38	−2.41^∗^
Economic Resources	4.73	1.48	4.82	1.47	.48
Mental Health	5.12	1.33	5.27	1.24	.97
IADLs	4.81	1.30	5.25	1.19	3.02^∗∗^
Physical Health	3.73	1.70	4.24	1.58	2.73^∗∗^
Social Resources	4.49	1.55	4.57	1.47	.42

^∗^ *P* < .05. ^∗∗^ *P* < .01.

**Table 3:** OARS resources latent variable measurement model results.

Construct/indicators	Loadings (*λ*)^a^	Uniqueness (*R* ^2^)	Loadings (*λ*)^a^	Uniqueness (*R* ^2^)
	T1^∗^		T2^∗∗^	
*Economic Resources*				
Sufficient Income	.81^b^	.66	.96^b^	.93
Overall Income	.70	.49	.61	.37
Meet Payments	.63	.40	.30	.09
*Mental Health*				
Exciting	.60^b^	.36	.53^b^	.28
Overall Mental Health	.53	.29	.49	.24
Life Satisfaction	.50	.25	.69	.47
IADLS				
Getting Out	.89^b^	.80	.94^b^	.88
Housework	.88	.78	.94	.88
Medicine	.65	.42	.77	.59
*Physical Health*				
Low Troubles	.70^b^	.49	.86^b^	.73
Overall Physical Health	.66	.44	.49	.24
Comparative Health	.46	.21	.48	.23
*Social Resources*				
Phone Talk	.64^b^	.41	.94^b^	.89
Visit Network Number	.47	.22	.08	.01
Visits With Others	.42	.17	.29	.08

^a^Parameter estimates are from the standardized solution. ^b^These indicator loadings were fixed to 1.0 (unstandardized) for model identification; all estimated loadings* P* < .01; except Time 2 Social Resources.

^∗^T1 Fit Indices: MLR *χ* ^2^ (*N* = 321; df = 80) = 144; CFI = .94; TLI = .92; RMSEA = .05; SRMR = .05.

^∗∗^T2 Fit Indices: MLR *χ* ^2^ (*N* = 201; df = 80) = 136; CFI = .93; TLI = .90; RMSEA = .06; SRMR = .07.

**Table 4:** Correlation Matrix of OARS Resources Latent Variables (Time1 below the diagonal; Time 2 above the diagonal).

	1	2	3	4	5
1. Economic Resources	—	.26^∗∗^	.43^∗∗^	.27^∗∗^	.08
2. Physical Health	.46^∗∗^	—	.15	.58^∗∗^	.14
3. Mental Health	.60^∗∗^	.83^∗∗^	—	.38^∗∗^	.25
4. IADLS	.21^∗∗^	.56^∗∗^	.42^∗∗^	—	.45^∗∗^
5. Social Resources	.27^∗∗^	.34^∗∗^	.35^∗∗^	.50^∗∗^	—

^∗∗^ *P* < .01.

**Table 5:** Correlation matrix of indicators for OARS measurement model (Time 1 below the diagonal; Time 2 above the diagonal).

Variables	1	2	3	4	5	6	7	8	9	10	11	12	13	14	15	16
(1) Age Group	—	−.18^∗∗^	.02	−.08	−.22^∗∗^	−.23^∗∗^	−.13	−.72^∗∗^	−.71^∗∗^	−.47^∗∗^	−.27^∗∗^	−.10	−.09	−.40^∗∗^	−.18^∗∗^	−.03
(2) Sufficient Income	−.05	—	.58^∗∗^	.28^∗∗^	.17^∗∗^	.18^∗∗^	.32^∗∗^	.27^∗∗^	.25^∗∗^	.12	.19^∗∗^	.30^∗∗^	.04	.14	−.02	.16
(3) Overall Income	.07	.56^∗∗^	—	.22^∗∗^	.07	.14	.21^∗^	.02	.04	−.01	.02	.16^∗^	.04	.08	.10	.09
(4) Meet Payments	−.04	.55^∗∗^	.41^∗∗^	—	.09	.06	.14	.12	.10	.06	.21^∗∗^	.10	.13	−.02	−.06	−.01
(5) Exciting	−.24^∗∗^	.22^∗∗^	.27^∗∗^	.20^∗∗^	—	.29^∗∗^	.35^∗∗^	.23^∗∗^	.17	.22^∗∗^	.05	.13	−.09	.14^∗^	.06	.20^∗∗^
(6) Overall Mental Health	−.08	.28^∗∗^	.31^∗∗^	.16^∗∗^	.27^∗∗^	—	.33^∗∗^	.16^∗∗^	.21^∗∗^	.07	.00	.33^∗∗^	.01	.07	.13^∗^	.03
(7) Life Satisfaction	−.10	.22^∗∗^	.24^∗∗^	.24^∗∗^	.38^∗∗^	.23^∗∗^	—	.19^∗^	.26^∗∗^	.20^∗^	.09	.18^∗∗^	−.02	.16^∗∗^	.04	.17^∗∗^
(8) Getting Out	−.69^∗∗^	.17^∗∗^	.11	.18^∗∗^	.33^∗∗^	.13^∗∗^	.20^∗∗^	—	.88^∗∗^	.73^∗∗^	.46^∗∗^	.32^∗∗^	.17^∗^	.40^∗∗^	.13	.02
(9) House Work	−.63^∗∗^	.04	.07	.16^∗∗^	.29^∗∗^	.11	.14^∗∗^	.79^∗∗^	—	.72^∗∗^	.48^∗∗^	.27^∗∗^	.16^∗^	.40^∗∗^	.07	.07
(10) Medicine	−.38^∗∗^	.19^∗∗^	.00	.02	.21^∗∗^	.03	.07	.55^∗∗^	.61^∗∗^	—	.38^∗∗^	.27^∗∗^	.14	.31^∗∗^	.07	.05
(11) Low Troubles	−.26^∗∗^	.19^∗∗^	.25^∗∗^	.21^∗∗^	.32^∗∗^	.26^∗∗^	.28^∗∗^	.41^∗∗^	.35^∗∗^	.30^∗∗^	—	.40^∗∗^	.41^∗∗^	.09	−.14^∗^	.00
(12) Overall Physical Health	−.16^∗∗^	.30^∗∗^	.29^∗∗^	.22^∗∗^	.33^∗∗^	.48^∗∗^	.20^∗∗^	.28^∗∗^	.24^∗∗^	.17^∗∗^	.45^∗∗^	—	.33^∗∗^	.16^∗^	.03	.08
(13) Comparative Health	−.28^∗∗^	.08	.12	.11	.25^∗∗^	.17^∗∗^	.13^∗∗^	.29^∗∗^	.27^∗∗^	.19^∗∗^	.35^∗∗^	.28^∗∗^	—	.10	−.13	−.03
(14) Phone Talk	.23^∗∗^	.04	.18^∗∗^	.03	.16^∗∗^	.08	.11	.35^∗∗^	.30^∗∗^	.20^∗∗^	.15^∗^	.20^∗∗^	.01	—	.07	.27^∗∗^
(15) Visit Network Number	−.21^∗∗^	.03	.07	.09	.11^∗∗^	.05	.04	.32^∗∗^	.21^∗∗^	.19	.10	.13	.09	.24^∗∗^	—	.19
(16) Visits With Others	−.03	.14^∗∗^	.05	−.01	.10	.09	.11	.10	.00	.08	.11	.09	−.01	.31^∗∗^	.25^∗∗^	—
Means	Time 1	*N* = 321	1.14	1.52	1.44	1.78	1.41	2.06	1.75	1.63	1.58	1.83	1.29	1.89	.86	2.41
	Time 2	*N* = 201	.99	1.52	1.42	1.72	1.42	1.97	1.77	1.55	1.47	1.77	1.17	1.83	.78	2.41
SD	Time 1	*N* = 321	.78	.78	.52	.50	.57	.70	.47	.46	.59	.40	.76	.77	.62	.85
	Time 2	*N* = 201	.83	.78	.77	.58	.57	.72	.49	.55	.67	.51	.78	.80	.61	.89

*P ≤ .05. **P ≤ .01.

## Appendices

### A. OARS Economic Resources

#### A.1. Sufficient Income

Are your assets and financial resources sufficient to meet emergencies?

1 Yes
0 No

Do you usually have enough to buy those little “extras,” that is, those small luxuries?

1 Yes
0 No

At the present time do you feel that you will have enough for your needs in the future?

1 Yes
0 No

#### A.2. Overall Income

Please tell me how well you think you are now doing financially as compared to other people your age—better, about the same, or worse?

3 Better
2  About the same
1 Worse

How well does the amount of money you have take care of your needs—very well, fairly well, or poorly?

3 Very well
2 Fairly well
1 Poorly

#### A.3. Meet Payments

Are your expenses so heavy that you cannot meet the payments, or can you barely meet the payments, or are your payments no problem to you?

0 Subject cannot meet payments
1 Subject can barely meet payments
2 Payments are no problem

### B. OARS Mental Health

#### B.1. Exciting

In general, do you find life exciting, pretty routine, or dull?

2 Exciting
1 Pretty routine
0 Dull

#### B.2. Overall Mental Health

How would you rate your mental or emotional health at the present time—excellent, good, fair, or poor?

3 Excellent
2 Good
1 Fair
0 Poor

#### B.3. Life Satisfaction

Taking everything into consideration how would you describe your satisfaction with life in general at the present time—good, fair, or poor?

2 Good
1 Fair
0 Poor

### C. OARS IADL

#### C.1. Getting Out

Can you use the telephone…

2 without help, including looking up numbers and dialing?
1 with some help (can answer phone or dial operator in an emergency, but need a special phone or help in getting the number or dialing)?
0 are you completely unable to use the telephone?

Can you get to places out of walking distance…

2 without help (drive your own car, or travel alone on buses, or taxis)?
1 with some help (need someone to help you or go with you when traveling)?
0 are you unable to travel unless emergency arrangements are made for a specialized vehicle like an ambulance?

Can you go shopping for groceries or clothes (assuming subject has trans.)…

2 without help (taking care of all shopping needs yourself, assuming you had transportation)?
1 with some help (need someone to go with you on all shopping trips)?
0 are you completely unable to do any shopping?

#### C.2. Housework

Can you prepare your own meals…

2 without help (plan and cook full meals yourself)?
1 with some help (can prepare some things but unable to cook full meals yourself)?
0 are you completely unable to prepare any meals?

Can you do housework…

2 without help (can clean floors, etc.)?
1 with some help (can do light housework but need help with heavy work)?
0 are you completely unable to do any housework?

Can you handle your own money…

2 without help (write checks, pay bills, etc.)?
1 with some help (manage day-to-day buying but need help with managing your checkbook and paying your bills)?
0 are you completely unable to handle money?

#### C.3. Medicine

Can you take your own medicine…

2 without help (in the right dose at the right time)?
1 with some help (able to take medicine if someone prepares it for you and/or reminds you to take it)?
0 are you completely unable to take your medicines?

### D. OARS Physical Health

#### D.1. Low Troubles

How much do your health troubles stand in the way of your doing the things you want to do—not at all, a little (some), or a great deal?

2 Not at all
1 A little (some)
0 A great deal

#### D.2. Overall Health

How would you rate your overall health at the present time—excellent, good, fair, or poor?

3 Excellent
2 Good
1 Fair
0 Poor

#### D.3. Comparative Health

Is your health now better, about the same, or worse than it was five years ago?

2 Better
1 About the same
0 Worse

### E. OARS Social Resources

#### E.1. Visit Network Number

How many people do you know well enough to visit with in their homes?

3 Five or more
2 Three to four
1 One or two
0 None

#### E.2. Phone Talk

About how many times did you talk to someone—friends, relatives, or others—on the telephone in the past week (either you called them or they called you?) (if subject has no phone, question still applies).

3 Once a day or more
2 2–6 times
1 Once
0 Not at all

#### E.3. Visits with Others

How many times during the past week did you spend some time with someone who does not live with you, that is, you went to see them or they came to visit you, or you went out to do things together?

3 Once a day or more
2 2–6 times
1 Once
0 Not at all
